# Why Do Hens Pile? Hypothesizing the Causes and Consequences

**DOI:** 10.3389/fvets.2020.616836

**Published:** 2020-12-10

**Authors:** Helen Gray, Rachel Davies, Ashleigh Bright, Ann Rayner, Lucy Asher

**Affiliations:** ^1^Asher Behaviour Lab, School of Natural and Environmental Sciences, Newcastle University, Newcastle upon Tyne, United Kingdom; ^2^FAI Farms Ltd., The Barn, Wytham, Oxfordshire, United Kingdom

**Keywords:** domestication, vortex behavior, collective behavior, one welfare, animal welfare, smothering, laying hen

## Abstract

Piling is a behavior in laying hens whereby individuals aggregate in larger densities than would be normally expected. When piling behavior leads to mortalities it is known as smothering and its frequent but unpredictable occurrence is a major concern for many egg producers. There are generally considered to be three types of piling: panic, nest box and recurring piling. Whilst nest box and panic piling have apparent triggers, recurring piling does not, making it an enigmatic and ethologically intriguing behavior. The repetitive nature of recurring piling may result in a higher incidence of smothering and could have unconsidered, sub-lethal consequences. Here, we consider the possible causes of recurring piling from an ethological perspective and outline the potential welfare and production consequences. Drawing on a wide range of literature, we consider different timescales of causes from immediate triggers to ontogeny and domestication processes, and finally consider the evolution of collective behavior. By considering different timescales of influence, we built four hypotheses relevant to the causes of piling, which state that the behavior: (i) is caused by hens moving toward or away from an attractant/repellent; (ii) is socially influenced; (iii) is influenced by early life experiences and; (iv) can be described as a maladaptive collective behavior. We further propose that the following could be welfare consequences of piling behavior: Heat stress, physical injury (such as keel bone damage), and behavioral and physiological stress effects. Production consequences include direct and indirect mortality (smothering and knock-on effects of piling, respectively), potential negative impacts on egg quality and on worker welfare. In future studies the causes of piling and smothering should be considered according to the different timescales on which causes might occur. Here, both epidemiological and modeling approaches could support further study of piling behavior, where empirical studies can be challenging.

## Introduction

Piling and smothering are aberrant behaviors in commercial laying hens. Piling occurs when hens crowd together in densely packed groups, and smothering refers to any mortality resulting from a piling event. Piling may result in smothering, but mortality is not always a consequence of piling (1). Bright and Johnson (2) defined three types of smothering: Firstly, panic smothers, or fear induced smothers. These occur as one-off events caused by disturbances and result in high levels of mortality (>20 birds). Secondly, nest box smothers occur when multiple birds use the same nest box. Nest box smothers can result in mortality of small (1 or 2) to large (>20) numbers of birds but are easily identifiable due to their location. The final category is creeping or recurring smothers (hereafter recurring smothers). Recurring smothers usually present as slow moving, seemingly non-panicked groups of birds. Recurring smothers typically result in mortality of smaller numbers of birds (1–10) but continue throughout the laying period and their cause remains unknown. Although the terminology in Bright and Johnson (2) specifically related to smothering, we use “nest box,” “panic” and “recurring” in this paper to also refer to piling events. Due to potentially differential causes and consequences of the three types of piling and smothering, the differentiation is important.

Whilst other problem behaviors, such as feather pecking, have been heavily studied, there has been limited research into piling and smothering. This may be in part due to the varied presentation of the behavior. Unlike feather pecking, piling is not easily defined or quantified and may differ between housing or breeds. For example, in the small number of papers describing piling, the definition of a pile is not consistent. Winter et al. (3) defined piling as “a cluster of mostly motionless laying hens standing in the closest possible proximity, with their heads mostly in the same orientation” with a stipulation that at least two birds must be involved. Piling has also been defined more numerically by some as “a minimum of 10 birds pressed against each other for at least 1 min” (1, 4). However, Herbert et al. [(5); under review] argue that this would lead to over-identification of piling such that they defined piling as “>30 tightly packed birds, to the extent that only the head and neck were visible, for 30 min”. In addition, where specific research into smothering exists, studies are often preliminary, on few flocks, or the information is gathered through self-reports from farmers. Despite the paucity of research in this area, we describe what literature there is below.

Bright and Johnson (2) described the three forms of smothering within a preliminary study of 10 commercial flocks. This study was the first to highlight smothering as a concern for commercial egg production, due to the impacts on a flocks' welfare and productivity (increased mortality and decreased egg production). This was followed up with a wider study exploring smothering more closely. Part One (6), concluded that smothering is common, with 60% of farmers experiencing smothering in their last flock, and 74% believing smothering to be a substantial issue, although no clear reduction strategies were identified. Part Two (7) investigated correlations between disease, housing, management practices and smothering. Nest box smothers were associated with nest box manufacturer (design) and breed of hens, whereas panic and recurring smothers (as a single category) were associated with nest box manufacturer, range use on a sunny day and the practice of feeding grit or grain on the litter. The authors considered smothering to be sensitive to delicately balanced bird, housing and management factors.

Smothering due to panic is a recently highlighted issue, however, nervousness and hysteria in laying hens has long been recognized. Hansen (8) described the “malady” of nervousness and hysteria in experimental caged and floor-raised hens, attributing effects of strain, social pressure (population density), pain and reduced environmental complexity. Panic can be attributable to specific events [e.g., unusual stockperson behavior; (9)] perhaps explaining the limited investigation into panic smothers. Nest box smothers have similarly had limited attention. Giersberg et al. (10) recently explored behaviors within, and use of, nest boxes by commercial and dual-purpose hens determining a breed effect on nest box smothers. However, the primary aim of this study was to compare the behavior of dual purpose and commercial laying hens rather than determine factors contributing toward smothers.

Recent studies of smothering have focused more specifically on the occurrence of piling. Campbell et al. (1) visually characterized piling from observations of the litter area of two commercial aviary flocks (>49,000 birds per flock; aviary divided into sections containing either 852 or 1,704 birds). Duration of piling events ranged from 1 to 359 min and 10 to ~229 hens participated. Average pile sizes showed approximately 4–5% of birds participated in piling. Piling occurred near the section dividers in early lay but this was not consistent in later observations. Piles were dynamic with birds leaving and joining, and some birds attempting to access the centre of the pile. Fewer than 7% of piling events were due to a disturbance, thus the majority of piling observations were aligned to the recurring smother definition (however no smothers were observed). Herbert et al. [(5); under review] observed more extreme events in a 12,000-bird flock (kept in 4,000 bird colonies) performing recurring piling, with up to 1,204 birds participating (average pile contained ~25% of the colony). In contrast to Campbell et al. (1), Herbert found piling to occur in the same location in 33/34 observations and found associations with environmental and bird-based parameters.

Whilst all types of piling have been understudied, the motivations underlying panic and nest box piling are more apparent compared to recurring smothering. In this review we specifically consider recurring piling events and suggest hypotheses for their causes and consequences, drawing from a wide range of literature. Recurring piling is of particular interest due to the apparent lack of trigger and the understudied welfare and production effects. This paper is intended to highlight areas of future research interest and to hypothesize on causes and impacts of piling, but by no means covers all possibilities and literature searching was not conducted systematically.

## Potential Causes

Behaviors can be induced by a range of factors over varying timescales, from immediate triggers to genetic influences. The generative mechanisms can be split broadly into proximate causes and ultimate causes, a concept first introduced by Mayr almost 60 years ago (11). Proximate causes concern the more immediate developmental, physiological or environmental triggers, whereas the ultimate causes concern evolutionary mechanisms of function and phylogeny. We use an adjusted version of Mayr's framework to review causes of piling behavior which occur at different time frames of influence both within and outside of a bird's lifetime. Specifically, we consider: (i) the timeline of events immediately preceding a piling event; (ii) the developmental stage of a hen's life; (iii) the effects of domestication, and iv) the evolution of collective behavior.

### Immediate Causes

**Figure 1:**
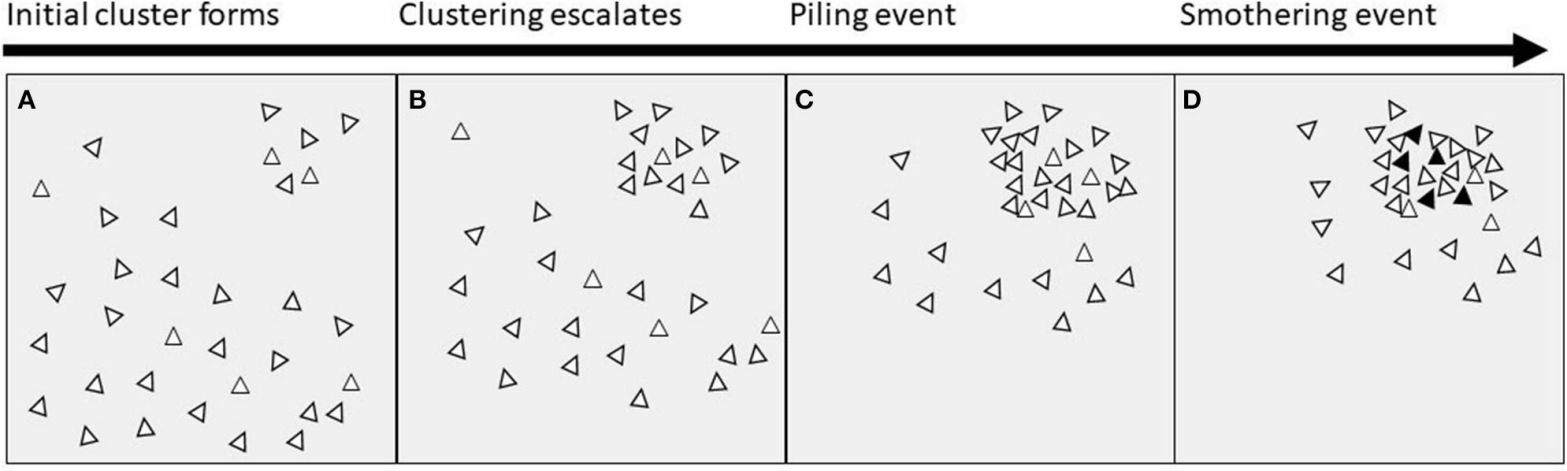
Proximate timeline of events leading to smothering. **(A)** An initial cluster of birds is formed by attraction to/repulsion from a stimulus or location, or by chance, or by timing factors; **(B)** the clustering behavior escalates due to social attraction, or attraction to/repulsion from the original stimulus or location; **(C)** a dense pile of birds is formed; **(D)** the pile exceeds an unknown threshold and ends in a smother, with mortality of birds indicated by black triangles.

The key feature of piling behavior is clustering of birds in one location, causing an extreme unevenness in distribution (Figure 1). We suggest that the initial unevenness could begin in one of the following ways: (i) birds being attracted to/repelled from a certain location or stimulus such as light; (ii) a routine or timing factor causing birds to gather in a particular space at a particular time; (iii) chance or randomness; (iv) or a combination of two or all of these. The clustering then escalates, causing more birds to join with the initial group, perhaps due to attraction to/repulsion from the initial stimulus or due to social factors (e.g., following birds already in the cluster). Piling behavior at some point tips into a smothering event, resulting in hen mortality. The threshold at which a pile turns into a smother is currently unknown and discussing potential reasons is beyond the scope of this paper.

#### Initial Cluster Formation

##### Light and temperature

Previous work shows that range use on a sunny day is positively associated with smothering events, with producers reporting that free range hens gather outdoors in sun spots (7). To our knowledge, this phenomenon has not been empirically studied in chickens and the reasons for the behavior remain unclear but may be driven by either attraction to heat or light or repulsion from shade or cold. Little is known about the thermal preferences of laying hens but producers identified daily temperature fluctuations as a potential cause of smothering (2) and clustering behavior changes dependent on temperature in white leghorn chickens (12). Although attraction to light (phototaxis) has not been experimentally shown in chickens, there is evidence of phototaxis in starling development (13) and in migrating nocturnal birds showing attraction to artificial light sources (14). There are also initial reports of piles being initiated by attraction to shards of light within a shed (3, 4). On a similar theme, some studies have found laying hens to have preferences for different light sources (15–17), light colors (18) and light intensities (19), with chickens spending more time in areas with their preferred light sources.

Birds have more photoreceptors than humans, allowing them to see into the UV light spectrum [around 100–400 nm wavelength; e.g., (20)]. Not only can birds detect wavelengths that we cannot, but also have better acuity in distinguishing colors. As such, in addition to considering the spectrum of light visible to humans (around 400–700 nm), we must also note that hens may be guided by attraction to, or repulsion from, wavelengths of light and color changes invisible to us.

Here we have highlighted light and temperature to exemplify attraction and repulsion to or from a stimulus, but it should be noted that attraction to other physical or auditory stimuli may play a role in the initiation of piling, especially as chickens have been shown to seek novelty (21). For example, Winter et al. (3) cite attraction to a novel food item as a cause of piling in Swiss flocks.

##### Location

Although smothering location has previously been considered unpredictable, Barrett et al. (6) and Herbert et al. [(5); under review] report consistencies in smothering and piling locations, respectively, suggesting that hens may be attracted to specific spaces. Similarly, Campbell et al. (1) reported approximately 86% of their observed piles to be located against a gate or wall. In a study unrelated to piling, some individual hens spent longer on the slatted area of the shed than the litter, although this was a tactic for hens in poorer condition to avoid pecks (22). By tracking hens, individual preferences have been shown for space use in aviary systems (23, 24) and for preferences in indoor and outdoor locations (25, 26). However, findings from another study suggested no evidence for general spatial preferences among laying hens (27). Location preferences in hens are therefore individual, and, if location is an important attractant in piling, it may be interlinked with daily time budgets and accessibility of spaces during the day.

##### Timing/Routine

Hens experience farm-specific daily routines, leaving particular times at which they are more likely to access the litter area and therefore more likely to pile. For example, feed is often provided consistently at the same times, farmers may walk through the sheds at specific points during the day, hens perch more in the evening and in the dark (28, 29) and are more likely to lay eggs in the morning [e.g., (30)]. A preliminary study on one flock showed that piling occurred consistently in the afternoon (5), suggesting a time-dependent component of the behavior, at a time when hens are not feeding, not laying or perching, nor disrupted by human presence. This observation is supported by research showing that open litter use can peak in the afternoon (31, 32), but it should be noted that the interaction of space use and time of day may also depend on chicken strain (33). Being aware of the times and spaces in which hens are likely to exhibit initial clustering behavior is the first step to combating the problem, even if the time or location are not themselves the primary cause.

##### Fear

The behavior described in some literature as “hysteria” or “panic” can lead to smothers which present differently from recurring piling [see **Introduction** and Richards et al. (9)]. Panic smothers typically form quickly and are thought to originate from disruptions which cause a contagion of fear or panic throughout the flock, or within a subgroup of the flock. Due to the slow-moving nature of recurring piling, it is considered not to be based entirely in panic [e.g., (1)], but components of the behavior may still be related to fear. Firstly, the initial cluster formation could be a result of a fearful response of one or more birds from an aversive stimulus and, secondly, the movement of birds toward conspecifics in the escalation stage may reflect a group response to perceived threat (see Cluster Escalation).

##### Randomness

Finally, initial clusters of birds may appear to be forming by random aggregation. Indeed, piles have been reported to occur when there are no obvious or discernible causes (1, 4). Randomness is a human-defined construct for the appearance of an outcome which we cannot explain. The movement and mechanisms that cause clusters would never be truly inexplicable but may be beyond what we are able to perceive or measure and thus may appear random. For example, the behavior may be guided by colors outside of our sensory capabilities (see Light and temperature) or by movements unseen by the human visual system but perceptible by the hens (20). Additionally, differences in air quality throughout the shed [e.g., aversive ammonia concentrations; (34)], or undetectable auditory stimuli [e.g., infrasound (35)] are also candidates for unexplained movements. Although perceived randomness should not be fully discounted as a theory of clustering, the previously mentioned producer opinion, preliminary studies and existing knowledge of hen behavior suggest that there may be measurable mechanisms which should serve as our starting points for the study of piling.

#### Cluster Escalation

Once a small cluster of hens has formed, the behavior escalates, such that more birds join and eventually form a pile. For this part of the process, we hypothesize that social attraction may be a key mechanism, in addition to, or in place of, the theory of spatial and stimulus attraction. Social attraction has been previously implicated in piling (3) and may be a key component of the behavior if the initial attractant is no longer visible or perceptible. The underlying theories of flocking behaviors suggest governance of collective movement by simple rules in individual behaviors. If an individual is too close to another, it moves away to avoid collision, if it is too far away, it moves toward its nearest neighbor (36). Piling behavior may follow similar rules, controlled by the position of neighboring conspecifics.

Previous results studying hens' attraction to flockmates are mixed. Laying hens in small groups can discriminate between pairs of their flockmates (37) but the evidence for flockmate preferences is unclear. One study found that hens choose to spend time with familiar conspecifics (38), whereas a later study provided no evidence for social preferences (27). Broilers have been shown to be socially attracted to one another, rather than socially averse (39) and similarly, free-range laying hens will move through pop holes in the same direction as conspecifics at a rate higher than would be expected by chance, suggesting a following mechanism (40). However, clustering in layers has also been found to be a function of motivation for resources rather than social attraction (41, 42). Even so, Lindberg and Nicol (43) found familiar birds will cluster more than unfamiliar birds and Odèn et al. (44) observed subgroups but only for those birds at the ends of sheds (i.e., location specific). Despite the conflicting data, social dynamics may be important in smothering as Campbell et al. (1) classified 60% of piling observations as being caused by “Hens on other side of gate appeared to interest hens in the focal section”. Interestingly, the social attraction which they speculate may have caused the pile, would also cause the recurring location of the pile (at the partition between groups of birds).

Along a similar vein, piling may be a product of synchronous behaviors, whereby hens engage in similar behaviors at the same time. For example, the initiation of feeding behaviors in broiler chickens can be reliant on the presence of other birds at the feeder (45). Synchrony can arise in a range of ways. One mechanism is response/social facilitation, whereby the behaviors involved in piling (such as slow circling) may be affected by the number of observable birds also engaged in the behavior. Social facilitation has been previously documented for preening, sitting and dustbathing behavior in hens (46). Local or stimulus enhancement is another potential mechanism, defined by the increased likelihood of visiting a location or stimulus by observing conspecifics in that space (47). Synchronization in group behavior can also increase as a reaction to a perceived threat, whereby group movement becomes more uniform [e.g., swimming speed in sticklebacks: (48)]. If initial cluster formation is perceived by hens as a reaction to a threat, then movements toward the initial cluster may result in synchronous behavior.

### Ontogeny/Developmental Causes

Piling during the laying period has been the focus of scientific literature to date. However, there is very little known about how piling behavior develops within the lifetime of a hen. In young chicks, clustering behavior is a frequently expressed functional behavior for thermoregulation and dense clusters of chicks are described as piles. Few studies have focused on piling and smothering during rearing but one study of pullet farms in Canada found smothering accounted for ~15% of mortality (49). The little data on smothering during the laying period indicate that smothering is worse at the peak of lay or just before (2, 6) whereas panic smothering is associated with hens <25 weeks of age (9). Whether the inception of recurrent smothering occurs during early life or not, there are several factors which could influence the development of piling within the lifetime of a hen.

It has been suggested that early life rearing conditions are potential risk factors for piling and smothering in adult hens (7, 50, 51). Indeed, early life and rearing conditions have influence on other problem behavior in poultry such as feather pecking (52–56). Developmental experiences during the rearing period and earlier have the potential to affect piling in a number of ways: (i) experiences during rearing could affect attraction or repulsion from stimuli or preferences for location; (ii) rearing environment could affect social behavior; (iii) early life adversity or stress could influence adult social behavior and stress responsiveness. We consider each of these in turn.

#### Rearing Impacts on Stimuli Preferences

It has long been known that preferences for environment and resources are determined by early life experiences. Given our suggestions in this review on stimulus attraction and repulsion as causes for piling, it is important to consider how early life may shape an individual's stimuli preference as an adult. For example, Dawkins (57) found that hens raised in battery cages preferred a battery cage over an outdoor run, whereas hens raised in a run preferred this over a battery cage. Conversely, previous experience seems not to be as important in litter preference, as birds at 29 weeks of age preferred litter to wire, even when raised on wire (58). Lighting at rearing leads to lighting preferences later in life (15) and rearing in a barren environment is known to affect spatial cognition and memory (59), both of which may be of importance given the implications of lighting and location in smothering.

#### Rearing Environment Impacts on Social Dynamics

In addition to influencing later life preference, the lighting environment during rearing can also affect propensity to perform social behavior. The light under which broilers are raised impacts synchrony, with brighter light resulting in more synchrony (60), although chicks have also been found to be more synchronized if brooded by a dark brooder compared to a heat lamp (61).

Social behavior could also be learned or influenced by early life experiences. Hens which cluster more as chicks may learn this has a positive association and retain clustering behavior as an adult. A lack of avoidance of conspecifics is a key characteristic of piling behavior. Chickens do not appear to avoid others until 6–10 weeks of age, around the time dominance hierarchies are typically established (62). It has been proposed that dominance hierarchies cannot be established in large groups of chickens (63). We could therefore speculate that the developmental stage of conspecific avoidance might be hampered by a lack of dominance hierarchies.

#### Stress Responses

Unlike some mammals, chicks have no stress hyporesponsive period, meaning they are responsive to stressors from when they hatch (64). It is proposed that either cumulative stress or mismatch between early life and later life stress can increase later stress responsiveness or fearfulness (65). In laying hens, effects of parental stress can influence levels of anxiety in the first 5 weeks of life (66). Early life stress in poultry has been found to increase later life stress responsiveness and fearfulness in chickens (67). Early life adversity is linked with development of other problem behavior in poultry e.g., feather pecking (52, 53, 66) and has been implicated in smothering (68). Key early life stressors might include noise from incubators, handling, lack of enrichment (69), vaccination (67) and transport stress (70), food and water deprivation (during transport or if chickens do not quickly locate feeder and drinkers in the rearing environment), or could be primed *in ovo*.

Enrichment provision during rearing is known to attenuate fear [e.g., (50, 71–74); reviewed in (75) and (76)] demonstrated that chicks with outdoor access at rearing were less fearful as adults. As such, enrichment at rearing may be of importance if fearfulness plays a role in piling. Fearfulness or stress responsiveness could be anticipated to either increase or decrease later piling behavior. If piling is a response to fearfulness then early increased adult fearfulness would increase piling behavior, however, it could also be the case that lack of avoidance of conspecifics is a response of hens who are low in fearfulness.

In summary, further investigation is required to understand the role of early life in the development of piling behavior, and could focus on the associations between the propensity to pile and: early life clustering behavior; early life stress; propensity to avoid conspecifics; and details of the rearing environment such as brooding, lighting or complexity.

### Domestication Processes

Domestication of a species involves selecting for traits beneficial for human-animal interactions, normally resulting in tamer and calmer individuals. The latest evidence suggests that the meat and egg-producing breeds of chickens familiar to us today were domesticated approximately 9,000 years ago from their wild ancestor, the red jungle fowl (77), though the time of initial domestication is contested.

In line with wild species being more reactive, red junglefowl have a heightened physiological and behavioral stress response compared with their domesticated counterparts. For example, jungle fowl show more fearful responses than domestic chickens in a range of behavioral tests (78–81). Fallahsharoudi et al. and Løtvedt et al. (82, 83) both show expression differences in stress-related genes of red junglefowl and white leghorn hens which suggest the attenuated response to acute stress in domesticated birds is result of changes in HPA axis-associated genome locations.

As well as differing reactions to stressful events, there are also some general differences in the behaviors shown between domesticated birds and jungle fowl. It is not that domestication has eliminated behavior from the commercial strains, but more that frequencies of behaviors and activity budgets vary. For example, Schutz and Jensen (78) found a commercial breed (white leghorn) to spend less time in higher energy activities such as social interactions and general movement, compared with red jungle fowl and a low selection breed (Swedish bantam). The white leghorn also ate from freely available food, whereas the red jungle fowl and bantam chose to eat from a diet mixed in with wood shavings, investing more time in their foraging activities. However, this study was carried out in a semi-natural forest location and so may not reflect the commercial strain's behavior in a more standardized setting. In novel situations, white leghorn birds stay closer to conspecifics, whereas jungle fowl tend to disperse and explore the environment more. This has been demonstrated both in chicks (84, 85) and in adults, with the results being more pronounced in adult females than males (86).

Overall, the literature indicates that domestication has attenuated acute stress responses with chickens remaining closer to conspecifics in novel situations. These characteristics may mean an initial cluster could form due to reduced exploratory behavior and/or increased interactions or attraction to flockmates. Reduced fearfulness or stress responsiveness could allow the cluster to escalate for two reasons: (i) hens are less likely to move away from an increasingly dense crowd of conspecifics; (ii) hens may flock together if they perceive the initial cluster to be formed as a fear or threat response. The effects of these behaviors are likely to be more pronounced in a production setting where large numbers of birds are housed together.

### The Evolution of Collective Behaviors

Piling is a collective behavior, where movement is deliberately or accidentally coordinated between individuals, and here evidence from other species may be relevant. Circular movements have been reported in the few studies which have observed piling (1, 5), with hens even moving over one another to reach the centre of a pile. Circling behavior of individuals around and toward a common center is known as vortex behavior and is performed by a wide range of taxa (from bacteria to humans) [reviewed by (87)]. Vortices are an emergent property of individuals following local rules, resulting in the collective output of the circular movements. These local rules are: attraction or repulsion forces to an external stimuli; turning behavior toward or away from the attractive or repellent stimuli; avoidance of other individuals, either because they form a physical barrier to movement, or short range avoidance (88). Delcourt et al. (87) considered five different causal factors involved in which vortex behavior: (i) attraction to a single stimulus concurrently; (ii) attraction prompted by the activity of conspecifics; (iii) a constraint surrounding individuals and limiting movement; (iv) repulsion from a surrounding stimuli [e.g., predation threat, known as the selfish herd; (89)]; (v) a social vortex caused by interactions between individuals. It is notable that the causes of a vortex are similar to those reviewed here on formation of an initial cluster and escalation of piling.

It seems likely that vortex behavior evolved as a byproduct of social behavior rather than conferring individual fitness benefit. Within some species and contexts vortex behaviors can be maladaptive, most famously exemplified by an ant mill where ants endlessly follow circular trails to their own detriment (90). The simplicity of the rules which lead to vortex behavior could explain why it is so widespread biologically and why it is exhibited even when it is maladaptive. Such maladaptive behavior could sustain in a population if it is rarely maladaptive or when animals encounter novel conditions which differ from their environment of evolutionary adaptedness (87), which could be the case for laying hens. With regard to other farmed species, salmon move in vortex behavior in sea cages (91). This is believed to have negative welfare consequences for heterogeneous groups of salmon where some individuals are unable to keep up with the speed of swimming of the majority (92). Heterogeneity between group members might be an important driver of vortex behavior with faster individuals beginning to circle around slower individuals (87). To conclude, piling could be considered as a maladaptive collective behavior, and, if observations of vortex behavior during piling are substantiated, the knowledge of the mechanisms of vortices could be used as a framework to further explore the drivers of this behavior in laying hens.

### Hypotheses

Based on the ideas and information synthesized above, we suggest the following, not mutually exclusive, hypotheses for the causes of recurrent piling behavior:

H1: Attraction or repulsion: a pile forms due to hens moving toward or away from an attractant or repellent.

H2: Socially influenced: a pile escalates due to social influences on behavior. This could be due to: (i) direct attraction toward other hens; (ii) attraction/repulsion to a stimuli which is more apparent due to behavior of other hens or, (iii) attraction to perform the same behavior as others.

H3: Influenced by early life experience: piling behavior will be influenced by early life experiences, by determining stimuli or social preferences or influencing fearfulness.

H4: Maladaptive collective behavior: piling could be described as maladaptive collective behavior because it is a vortex behavior which has negative individual fitness consequences.

In future studies the causes of piling and smothering should be considered according to the different timescales on which they might occur and we propose testing of the hypotheses above to elucidate the causes of smothering.

## Consequences

Aside from obvious cases of mortality, there is currently little known about the welfare and production consequences of piling, as well as the impacts on the farm workers. In this section, we present some potential consequences based on the characteristics of piling behavior, the effects of known stressors on hen health and egg quality, and the literature of producer mental health and well-being.

### Welfare and Health Consequences

Piling and smothering involve large numbers of birds occupying relatively small spaces which we hypothesize could lead to heat stress, physical injury and increased fear or stress. Two preliminary studies have so far found indicators of heat stress (5) and negative affective state during piling events (unpublished data from LA's group).

#### Heat Stress

The thermoneutral zone refers to ambient temperatures in which an endotherm can maintain core body temperature without exceeding energy use above its basal metabolic rate. In chickens, the thermoneutral zone is reported as between approximately 17–25°C [e.g., (93)] and at temperatures above this, the birds must actively cool themselves. Chickens are not able to sweat and are insulated by their feathers, making heat loss more challenging. A chicken's cooling mechanisms involve heat exchange via the wattle and comb, and active behaviors such as panting and wing spreading. There is some evidence that domestication may have made commercial breeds more susceptible to the effects of heat stress than are red jungle fowl (94). Add to this that the number of birds in a piling event can reach 180 birds per m^2^, piles can last for a number of hours and can occur daily (5) and heat stress becomes a justifiable welfare concern.

Although the temperature in a piling event is unknown, previous literature gives insights into physiological outcomes of heat stress [see (95) for a review]. Studies which experimentally induce heat stress implement a range of temperatures (often above 30°C) and measure physiological and/or behavioral changes. For example, acute heat stress in laying hens (38°C for 140 min) causes decreases in arterial blood carbon dioxide and blood bicarbonate, and increases lactate and blood pH (96). For broilers, 5 week old birds exposed to temperatures of 32°C for 6 h showed signs of oxidative stress (97) and broilers exposed to 36 and 38°C exhibited significant increases in body temperature and changes in breathing rate (98). Physiological studies from the 1940s suggest that, for white leghorn laying hens, body temperature cannot be controlled when environmental temperatures reach approximately 40.5°C, after which point mortality occurs (99). Squibb et al. (100), however, found that hens could withstand temperatures of 44°C, with this attributed to the hens experiencing a wide range of diurnal temperatures. The impacts of heat stress depend also on other environmental parameters, such as humidity, as well as the previous acclimatization of the bird (101), and the breed (102), all of which should be considered for the study of piling consequences.

Chronic heat stress in laying hens has been shown to result in decreased body weight, reduced food intake, inhibited immune function [(103); 35°C for 5 weeks] and reduced liver weight [(104); 32.6°C for 14 days]. This is potentially of interest when considering the recurring nature of piling events, whereby hens may be exposed to increased heat multiple times a week. However, the cyclic variations in temperature which may be experienced with piling, would provide the hens with time to recover, in contrast to true chronic exposure [e.g., (103)]. Indeed, Sykes and Fataftah (105) demonstrated that intermittent exposure to heat stress acclimatized laying hens to cope with high temperatures (38°C).

#### Physical Injury

Due to the density of individuals involved it is plausible that birds suffer injuries such as scratches, feather damage or bone damage as a result of piling. Laying hens are susceptible to bone damage and particularly to the keel bone (a protruding extension of the sternum) (106). The welfare impacts of keel bone damage are well-documented [see Riber et al. (107) for a review] and include physical pain (108), depressive-like states (109) and changes to the hens mobility (110). In non-cage systems, traumatic keel bone fractures have been suggested to arise from collisions with drinkers and perches [see (111)]. It is possible that hens in the centre or at the bottom of a pile may experience a similar amount of force required to cause a fracture. Recent evidence highlights that external force caused by collisions may not be the main cause of keel bone fractures (112), however this does not preclude the possibility for piling to result in keel bone fractures. Other bones could also be affected by piling, such as breaks in the humerus, though these types of injuries are less well-studied in laying hens and are more often linked with breaks caused at depopulation and processing [e.g., (113)]. Future research into the potential for injuries resulting from piling could initially focus on keel bones since fractures from traumatic impacts are apparent through palpation, rather than requiring radiographs or dissection. We note, however, that palpation cannot detect all instances of keel bone damage, but that palpation would present a non-invasive starting point to study the physical impacts of piling [see (114)].

#### Behavioral and Physiological Stress Effects

Aside from the potential adversities of heat and physical injury, the performance of piling behavior may itself be stressful. However, the paucity of data on piling means that there is a high degree of uncertainty around how negative consequences could present. We may expect to see responses similar to those in other stress-inducing situations, including increased corticosterone levels [e.g., (115)] and behavioral responses such as increased feed and water intake (116) or deviations from a baseline level of activity.

Frequent exposure to stressors (as may occur in recurrent piling) causes stress overload, which is more likely in cases where the predictability or controllability of the stressors is lower (117). Therefore, if piling is found to be unpredictable or uncontrollable, the effect on the hens may be greater.

Stressors also have downstream effects on immunity. For example, stress increases intestinal norepinephrine levels which, in turn, stimulates the growth of several bacterial species and increases abundance and pathogenicity of *Escherichia coli* (118). The results can lead to issues such as *E. coli* peritonitis syndrome, which can cause acute mortality, up to 15% above normal levels (119). In addition, increased levels of harmful bacteria unbalance the gut microbiota and can leave the individual open to colonization with further harmful foodborne pathogens like salmonella and campylobacter (120), posing a potential food-safety risk to consumers. If piling is stressful, it could lead to gut problems that past literature has not considered, and may pose potential health risks for both hens and humans.

Finally, the timescale over which stress responses are measured should be considered. For example, the effects of acute stress in domesticated chicken species, although initially not as pronounced as in their progenitor species, may be longer lasting. For example, Ericsson et al. (81) found that red jungle fowl had a more heightened initial stress responses to a restraint test, but returned to baseline levels more quickly. Domesticated species did not return to baseline within the hour test period. As such, if piling is found to be a stressful event, then effects may be prolonged, even once the pile is dispersed.

### System Considerations

While piling and smothering events could occur in all systems (121), they have the potential to be more problematic in cage-free flocks where larger numbers can pile together than in caged systems. Flock size in cage-free production has increased over the last 15 years with >30,000 bird flocks in the EU (predominantly barn or aviary) and 12–16,000 bird flocks in the UK (predominantly free-range; kept in 4,000 bird colonies). Group size and stocking density are associated with the incidence of other problematic laying hen behaviors such as injurious feather pecking and aggression (122). If, as hypothesized in this review, piling and smothering are associated with social preferences or stimuli, they are potentially more difficult to manage, and the consequences more severe, as flock size increases.

Furthermore, as flock sizes increase, the majority of newly built houses are “multi-tier” systems (as these systems house more birds in the same footprint as a single deck system) and are becoming more prevalent in barn and free-range egg production (pers comm, D Brass). Cage-free housing design affects hen movement, behavior, and welfare [e.g., (123–125)] and nest box smothers (7). Understanding how multi-tier systems impact upon piling and smothering will be important, particularly as the global egg industry transitions to cage-free production.

### Production Consequences

Farm viability, efficiency and profitability are important factors in sustainable production. In laying hen systems, a fine balance exists between resource inputs (e.g., labor, birds, feed, medicines, housing) and production outputs (eggs). Piling and smothering have potentially important implications for egg production because of increased mortality and reduced egg quantity and potentially quality. Bright and Johnson (2) reported smothering to cause between 0.37 and 10.5% mortality in 10 flocks, with estimates of 1.96–61.8% total egg loss. These production impacts may be significant for the egg sector where, in the UK for example, the average egg price to producers fell by ~10 pence per dozen between 2015 and 2019 and, even though egg price is higher now in 2020, margins are still poor (126, 127).

#### Mortality

Mortality in hens has a significant negative effect on the efficiency of a flock by reducing the input:output gains (128). For instance, increased mortality will increase the input of electricity per bird (for light, ventilation, feed etc.) as the laying cycle progresses (129), as well as decreasing the total egg yield over a flock cycle. The result is a reduced margin per bird and an increased environmental impact, reducing the economic viability of the flock and increasing the emissions per bird (129). We anticipate that flocks with recurrent piling may have higher cumulative mortalities due to direct mortalities from smothering, and additional mortality from indirect health impacts (see Behavioral and Physiological Stress Effects).

#### Egg Quality

Class B eggs are paid at a fraction of the price of Class A, which means preserving shell quality through good management and limiting stress factors is key in maintaining profitability of a flock. Issues with shell quality comprise 80–90% of egg quality issues, making it a key concern for producers (130). There is, as far as the authors are aware, no research which has studied the impact of piling on egg quality. Here we present ideas on how egg quality may be impacted, based on relevant evidence from other areas of poultry science.

A single stressful disturbance, such as a smothering or piling event, could be enough to affect the synchronization of the egg formation and cause egg-quality faults such as thin-shelled or soft eggs (131). Heat stress (discussed in Heat Stress) also causes weak shells, as reactionary panting behavior can result in changes in acid-base status and a loss of carbon dioxide needed for shell formation [see (132)]. In addition, physical injury as a result of overcrowding can result in body-checked eggs or flat-side eggs, particularly if the incidence occurs as the shell begins to form in the oviduct (133, 134). Overcrowding can also increase the incidence of shell texture defects such as rough shells (135). As the shell is the first defense against pathogen penetration, any defect of shell quality has the potential to also be a food-safety risk (136).

When shell quality deteriorates, incidences of shell breakages along the egg packing belt can increase and result in contamination of otherwise good quality eggs. Contaminated eggs then become downgraded to Class B as legislation states that “Class A eggs shall not be washed or cleaned, before or after grading” (137). To combat this, producers run belts at a slower speed, meaning packing time increases with the further knock-on effect of increased cleaning time, ultimately leaving less time for other tasks both within the poultry shed, and in other areas of the business.

Finally, as covered in Section Physical Injury, we anticipate that birds involved in a piling event may experience physical injury in the form of keel-bone fractures. Nasr et al. (110), found that birds with keel fractures had lower production and lower egg weight than those without. The difference could be due to the impact of pain or stress on the reproductive hormones, as elevated corticosterone suppresses follicular development (138).

The true financial impact of egg quality issues is difficult to measure as a commercial average, and will be impacted by flock-specific factors, such as age, bird numbers and current health status, and farm factors such as contract type. Flock- and farm-specifics aside, we anticipate that any stress from piling events could have the potential to negatively impact egg quality, and result in economic losses to the producer.

### Worker Welfare

The unpredictability of working with stock is an intrinsic source of stress to farmers and farmworkers. Stock-crises such as smothering or piling events can have a knock-on effect on often inflexible workdays and impact the long-term profitability of the farm (139). As discussed, piling events have the potential to impact immunity and increase disease prevalence in a flock, as well as impacting egg quality. The financial implications of this could include; production loss through mortality, dead stock disposal costs, antibiotic cost and other associated veterinary fees. Previous research indicates that worries surrounding finances are the most important stress factor to farmers' lives, with time pressures being second to this (140). Sustained behavioral and production problems within a flock therefore have the potential to more severely impact producer well-being than do singular disturbances. Poorer mental health may be a product of the overall duration of the issue and/or the cumulative negative impacts of exposure to bird mortality and decreased production. Farmer well-being and animal welfare are bi-directionally linked and therefore it is prudent to consider both of these when discussing sustainable and responsible food production (141).

### Hypotheses/Predictions

Based on the literature discussed above, we present four hypotheses on the consequences of recurring piling event:

H1: Welfare impacts: recurrent piling events will negatively impact animal welfare through one or more of the following: (i) heat stress; (ii) physical injury; (iii) stress

H2: Production impacts: recurrent smothering in flocks will cause reduced production through (i) mortality and/or (ii) reduced egg quality, and their knock-on consequences

H3: Worker welfare: recurrent piling will be detrimental to the mental well-being of farm staff through (i) exposure to increased mortality; (ii) decreased production and/or (iii) unpredictability of piling events.

H4: System effects: recurrent piling and smothering will occur more frequently as group size and stocking density increases because: (i) larger numbers of birds are able to pile together; (ii) birds have social stimuli and social preferences more likely to result in abnormal behaviors.

## Conclusions

This review has synthesized a range of literature to provide ideas and hypotheses on the potential causes and consequences of recurring piling events in laying hens. To mitigate against piling will require at least some understanding of the biological causes, which we acknowledge may take time. The causes are also potentially multifactorial which suggests that different prevention strategies would be necessary for successful mitigation. For example, we identified that over different time scales the same factors repeatedly appeared, such as socially motivated behaviors and modulations in stimulus attraction. If stimulus and social attraction are indeed causes of piling, they may need to be assessed at different life stages to prevent the onset of the behavior. An epidemiological approach could support the study of the causes of at different timescales.

Two interesting questions raised by this review relate to fearfulness and the smothering threshold. Firstly, what is the role of fearfulness in piling? Is fearfulness in piling flocks lower, allowing greater concentrations of birds, or is it higher, leading to groups forming to reduce fear? Secondly, what causes piling to become smothering? Either smothering is a simple escalation in piling behavior whereby densities result in death, or there is a change in piling behavior which leads to mortalities. Questions such as these may benefit from modeling approaches to further narrow down potential mechanisms and aid empirical studies. The consequences of piling, both at flock- and farm-level should also be a focus of future research, such that the true impacts can be understood and managed.

## Data Availability Statement

The original contributions generated for this study are included in the article/supplementary material, further inquiries can be directed to the corresponding author/s.

## Author Contributions

All authors wrote sections of the manuscript. HG produced Figure 1. All authors contributed to manuscript revision, and read and approved the submitted version.

## Conflict of Interest

The authors declare that funding for project BB/T001747/1 which funds LA, HG, and RD is in part contributed to by The Lakes Free Range Egg Company Ltd. Relevant commercial information was sought and feedback received from The Lakes Free Range Egg Company, but the content or presentation of the paper was not altered. The authors also work on other collaborative projects with egg producers and other partners with a vested interest in the poultry sector. However, the goal of all authors was to present the current evidence, thus the content of the paper was unaffected by these relationships.
